# Stromal-to-Epithelial Transition during Postpartum Endometrial Regeneration

**DOI:** 10.1371/journal.pone.0044285

**Published:** 2012-08-27

**Authors:** Cheng-Chiu Huang, Grant D. Orvis, Ying Wang, Richard R. Behringer

**Affiliations:** 1 Program in Developmental Biology, Baylor College of Medicine, Houston, Texas, United States of America; 2 Department of Genetics, University of Texas M.D. Anderson Cancer Center, Houston, Texas, United States of America; 3 Center for Stem Cell and Developmental Biology, University of Texas M.D. Anderson Cancer Center, Houston, Texas, United States of America; 4 Program in Genes and Development, University of Texas, Graduate School of Biomedical Sciences at Houston, Houston, Texas, United States of America; Brigham and Women's Hospital, United States of America

## Abstract

Endometrium is the inner lining of the uterus which is composed of epithelial and stromal tissue compartments enclosed by the two smooth muscle layers of the myometrium. In women, much of the endometrium is shed and regenerated each month during the menstrual cycle. Endometrial regeneration also occurs after parturition. The cellular mechanisms that regulate endometrial regeneration are still poorly understood. Using genetic fate-mapping in the mouse, we found that the epithelial compartment of the endometrium maintains its epithelial identity during the estrous cycle and postpartum regeneration. However, whereas the stromal compartment maintains its identity during homeostatic cycling, after parturition a subset of stromal cells differentiates into epithelium that is subsequently maintained. These findings identify potential progenitor cells within the endometrial stromal compartment that produce long-term epithelial tissue during postpartum endometrial regeneration.

## Introduction

The mammalian uterus is the site of embryo implantation, placentation, and fetal development. This reproductive organ is also considered a common origin of diseases [1]. For example, endometriosis [2] and endometrial cancers [3] are the two most prevalent gynecological conditions that occur in millions of women of reproductive age worldwide. These gynecological syndromes can lead to severe pelvic pain, infertility, poor quality of life and death. The cellular mechanisms that lead to the development of these pathologies are still unclear.

The endometrium undergoes cyclic cell proliferation and programmed death controlled by rhythmic fluctuations of the ovarian hormones during the estrous cycle [4], [5] ( Figure S1). Mammalian females experience hundreds of estrous cycles during their reproductive lifetimes. Thus, although standard histological analysis gives the impression of a static tissue, the endometrium is in reality a highly dynamic uterine tissue compartment that experiences a significant amount of cell turnover during homeostasis. In humans, many anthropoid primates, phyllostomid and molossid bats, and elephant shrews, a significant portion of the endometrium is shed during menstruation [6], [7]. The cell turnover during the estrous cycle in non-menstruating species and regenerative events in menstruating species and postpartum regeneration in both types of species may rely on similar or different mechanisms.

The regenerative ability of the endometrium suggests that somatic stem or progenitor cells play an important role in both uterine homeostasis and regeneration. Somatic stem cells are multipotent progenitors that can give rise to a variety of different cell types. They can maintain physiological homeostasis within a tissue and initiate the regeneration process in response to tissue damage [8]. Recent progress in the identification of quiescent somatic stem cells has suggested that they also exist in the human and mouse endometrium [9]; however, little is known about their in vivo behaviors during uterine homeostasis and regeneration.

The endometrium contains two epithelial cell populations, the luminal epithelium (LE) that lines the lumen of the uterus and the glandular epithelium (GE) of the endometrial or uterine glands that secrete factors required for embryo implantation and conceptus development [10]. Stromal cells compose an additional cell population of the endometrium that separates the LE and GE from the myometrium. The LE and GE are thought to derive from fetal Müllerian duct epithelial cells; whereas the stroma is thought to derive predominantly from the mesenchyme surrounding the Müllerian duct [11]. Both endometrial epithelial and stromal compartments contain BrdU label-retaining cells [12], [13]. These cells could serve as stem/progenitor cells for homeostasis and regeneration.

We have investigated the cellular mechanisms that regulate endometrial homeostasis and postpartum regeneration, using genetic fate mapping in the mouse. Our findings suggest that during homeostasis the epithelial and stromal tissue compartments are maintained by cells within each compartment. However, after parturition a subset of stromal cells differentiates and incorporates stably into the luminal and glandular epithelial compartments. These findings suggest that the tissue compartments in the adult uterus exhibit different behaviors during homeostasis and regeneration. The parturition-induced stromal-to-epithelial transition may have implications for uterine pathologies.

## Results

### Cellular mechanisms of postpartum endometrial regeneration in the mouse

**Figure 1:**
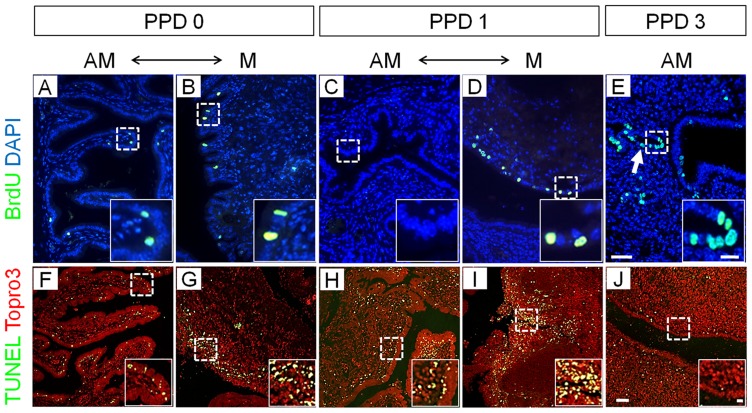
Cellular dynamics during endometrial regeneration after parturition. Cell proliferation assayed by BrdU labeling (A–E) and cell death assayed by TUNEL (F–J) in the postpartum endometrium. AM: anti-mesometrial region of uterus; M: mesometrial region; PPD 0, 6 hours after parturition; PPD 1, 30 hours after parturition; PPD 3, 72 hours after parturition. Boxed areas are high-magnification images from highlighted zones in A to J. Scale bars  = 50 µm in A–J; 10 µm in all boxed areas.

To investigate the cellular mechanisms of regeneration in the postpartum endometrium, we first examined cell proliferation and cell death in wild-type mice. Immediately within six hours after parturition (postpartum day 0, PPD 0; n = 3), there were few proliferating stromal cells and no proliferating LE cells detected on the anti-mesometrial side (Figure 1A), whereas a small population of LE cells located on the mesometrial side of the uterus, the placentation locus, was labeled by BrdU immunoreactivity (Figure 1B). Within 24 hours after parturition (postpartum day 1, PPD 1; n = 3), BrdU positive cells were predominantly found in the LE on the mesometrial side while anti-mesometrial tissue remained quiescent (Figure 1C and 1D). Within 72 hours postpartum (postpartum day 3, PPD 3; n = 3), cell proliferation was detected in the LE but predominantly in the GE and very little in the stroma (Figure 1E).

Concurrently, at PPD 0 and 1 (n = 3 for each experimental group), TUNEL analysis revealed significant levels of cell death in LE and stroma of the mesometrial and anti-mesometrial regions (Figure 1F–I). TUNEL-positive signals were more prominent in cells between the LE and deeper layer of stromal cells in both mesometrial and anti-mesometrial regions. At PPD 3 (n = 3), only a few TUNEL-positive cells were identified in the stromal region closest to the uterine lumen (Figure 1J). Thus, there are regional and temporal changes in cell proliferation and cell death in the endometrium after parturition.

### Endometrial epithelium and stroma maintain cell identity during homeostasis

**Figure 2:**
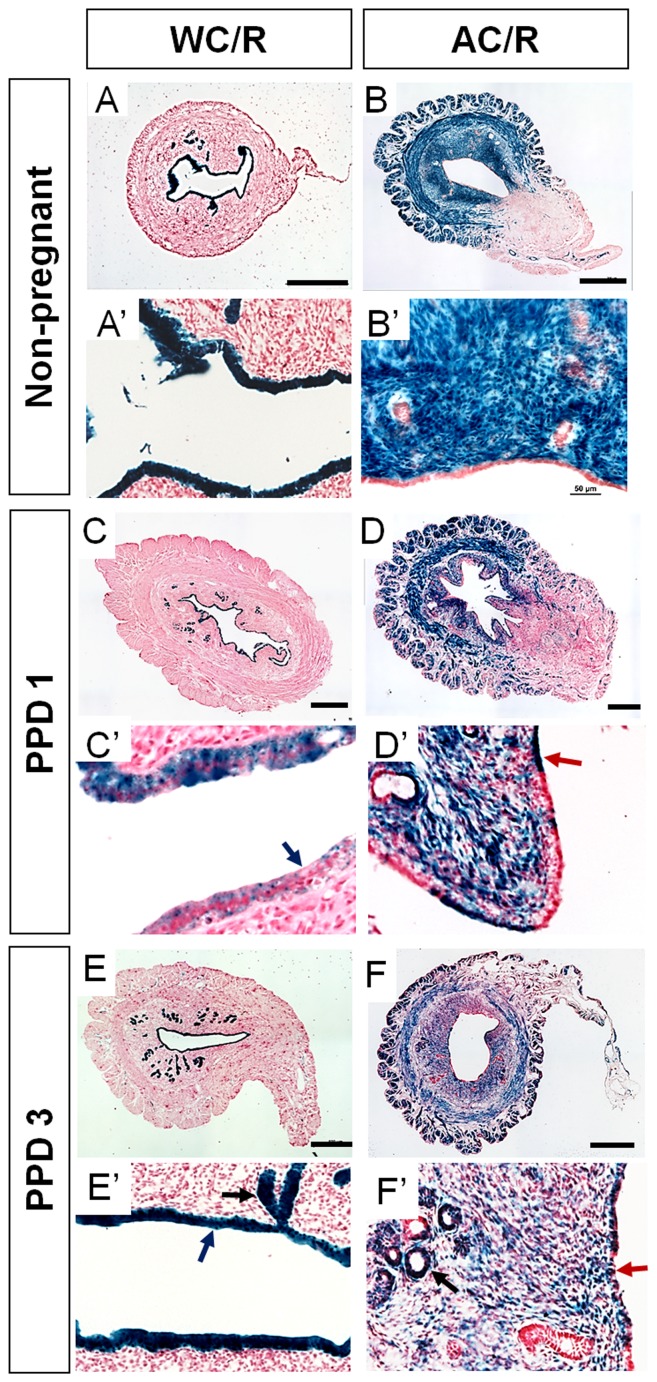
Stromal-to-epithelial transition in the postpartum endometrium. β-gal stained uterine cross sections from non-pregnant and postpartum (PPD 1 and 3) *Wnt7a-Cre; R26R-lacZ* (*WC/R*) and *Amhr2-Cre; R26R-lacZ* (*AC/R*) mice. (A and A′) Non-prenant WC/R female (n = 5). All luminal and glandular epithelia are β-gal positive but the stroma and myometrium are negative. (B and B′) Non-pregnant AC/R female (n = 5). The stroma is β-gal positive but the epithelia are negative. (C and C′), PPD 1 in WC/R mice (n = 4) is similar to the non-pregnant WC/R female except that there are regions of the luminal epithelium that are β-gal negative (blue arrows). (D and D′), PPD 1 in AC/R mice (n = 5) is similar to the non-pregnant female except there are regions of the luminal epithelium that are β-gal positive (red arrow). (E and E′), PPD 3 in WC/R mice (n = 4) appears the same as non-pregnant, only luminal and glandular epithelial tissues are β-gal positive. (F and F′), PPD 3 in AC/R mice (n = 5) appears similar to PPD1 with stroma-derived luminal epithelium (red arrow) except that there are also stroma-derived glandular epithelium (black arrow). Scale bars  = 500 µm in A–F; 50 µm in A′–F′.

Site-specific DNA recombinases (e.g. Cre) have been used for genetic fate-mapping studies to identify progenitor cells that give rise to various cell types [14]. Tissue-specific expression of these recombinases coupled with recombinase-activated cellular reporter mouse strains (e.g. *lacZ*) catalyzes a permanent genetic change that leads to persistent reporter expression in all subsequent cellular progeny regardless of their differentiation state. *Wnt7a* expression marks the Müllerian duct epithelium and postnatal LE but not GE [15]. To follow the fate of the epithelial compartment of the adult uterus we generated a *Wnt7a-Cre* bacterial artificial chromosome transgenic mouse line (Figure S2) and bred it with the *R26R lacZ*-based Cre reporter mouse strain [16], [17]. The uteri of non-pregnant virgin *Wnt7a-Cre*; *R26R* double heterozygous females (n = 5) between 2- to 6-months of age with active estrous cycles were examined for *lacZ* expression. β-galactosidase (β-gal) staining was detected only in the LE and GE and never in stromal or myometrial cells (Figure 2A and 2A′). Cre reporter expression in the GE is consistent with the idea that the GE is derived from the LE [10].

*Amhr2* expression is detected in the mesenchyme surrounding the Müllerian ducts and subsequently in the myometrium but not the stroma of the adult uterus [18]–[20]. It is thought that the Müllerian duct mesenchyme gives rise to the stromal and myometrial compartments of the adult uterus. To follow the fate of the stromal compartment of the adult uterus during homeostasis and postpartum regeneration we used an *Amhr2-Cre* knock-in mouse line. The *Amhr2-Cre* allele is active in the mesenchyme of the Müllerian duct [21]. The uteri of non-pregnant virgin *Amhr2-Cre*; *R26R* double heterozygous females (n = 5) between 2- to 6-months of age with active estrous cycles showed β-gal staining restricted to endometrial stromal and myometrial cells and never in the LE or GE (Figure 2B and 2B′). The above results suggest that during homeostasis (i.e. the estrous cycle) the epithelial and stromal compartments of the uterus maintain their respective fates and that cell replacement are mediated by epithelial- and stromal-restricted cells.

### Genetic fate-mapping shows stromal-to-epithelial transition during postpartum regeneration in the mouse endometrium

We next examined the fate of the epithelial and stromal compartments of the uterus after parturition when the endometrium is under the regenerative process. At PPD 1 in the *Wnt7a-Cre*; *R26R* uterus (n = 4), β-gal expression was limited to the LE and GE (Figure 2C). Interestingly there were discrete regions in the LE that were β-gal negative (Figure 2C′, blue arrow), suggesting the possibility of derivation from non-*Wnt7a-Cre*-expressing cell types. At PPD 3 (n = 4), β-gal expression was detected in the entire LE and GE (Figure 2E and 2E′). Thus, resident epithelial cells maintain their fates as they contribute to endometrial regeneration after parturition.

Interestingly at PPD 1 in *Amhr2-Cre*; *R26R* females (n = 5), we observed β-gal expression in stromal cells similar to the pattern in non-pregnant cycling females but also patches of β-gal expressing LE and GE (Figure 2D), suggesting that a subset of stromal cells had changed fate to contribute to the epithelial compartment of the endometrium. The *Amhr2-Cre* derived stromal cells that incorporated into the postpartum LE exhibited epithelial cell morphology (Figure 2D′). We also found this same mosaic pattern of stromal-to-epithelial transition in the endometrium of *Amhr2-Cre*; *R26R* females at PPD 3 (Figure 2F; n = 5). The stroma-derived cells were also capable of becoming GE (Figure 2F′, red arrow). We discovered that about 50% of endometrial glands in these females were mosaic for β-gal expression (Figure 2F′, yellow arrow), suggesting that individual uterine glands are likely derived from more than one progenitor cell.

### Stroma-derived endometrial cells in the epithelial compartment express cytokeratin

**Figure 3:**
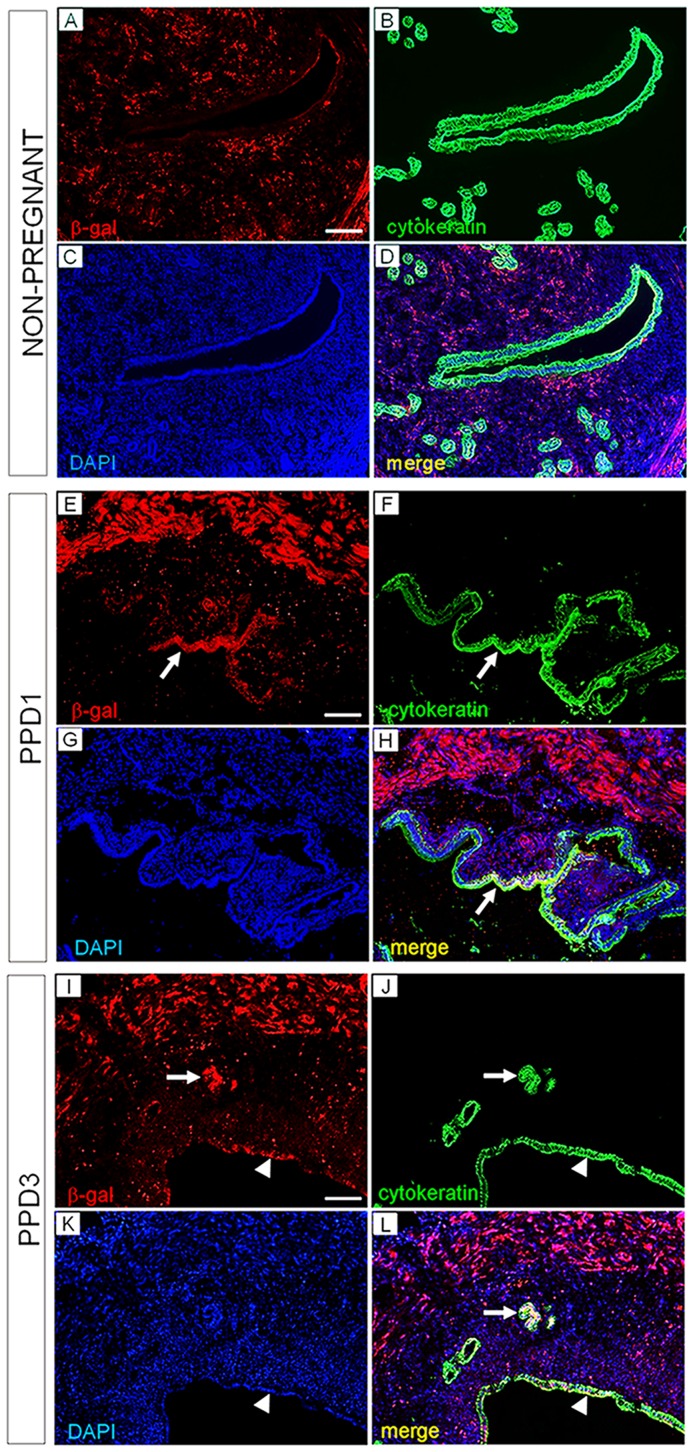
Stroma-derived luminal and glandular epithelium express the cytokeratin epithelial marker. β-gal and cytokeratin double immunofluorescence labeling of *Amhr2-Cre*; *R26R* virgin and postpartum endometrium. (A–D) In non-pregnant uteri, β-gal immunoreactivity is specifically detected in the endometrial stroma and muscle cells. Luminal epithelial (LE) and glandular epithelial (GE) cells are labeled with cytokeratin immunofluorescence (E–H) In PPD 1 uteri, strong β-gal immunosignal is detected in the LE (white arrows) and co-localize with cytokeratin staining (I–L) At PPD3, β-gal-marked stroma-derived GE and LE cells express cytokeratin (white arrows: GE; white arrow heads: LE). Scale bars  = 50 µm.

The molecular character of the stroma-derived LE and GE was examined by dual immunofluorescent staining for β-gal and cytokeratin, an epithelial differentiation marker of the endometrium. In non-pregnant *Amhr2-Cre*; *R26R* uteri, β-gal positive immunoreactivity was detected in stromal cells and myometrium but not in LE and GE. (Figure 3A–D). At PPD 1, β-gal positive stroma-derived LE cells expressed cytokeratin (Figure 3E–H, arrows). Subsequently, at PPD 3, β-gal positive stroma-derived GE and LE cells both expressed cytokeratin (Figure 3I–L; arrows: GE, arrow heads: LE). Thus, after parturition, stroma-derived cells in the LE and GE acquire a differentiation state characteristic of epithelium.

### Expression of *Amhr2* is restricted to myometrium in adult mice and not induced by parturition or during endometrial regeneration

Our genetic fate-mapping system suggesting a stromal-to-epithelial transition after parturition depends on correct regulation of the *Amhr2* locus for tissue-specific Cre expression. Although *Amhr2* expression in the adult uterus is limited to the myometrium [20], it is formally possible that it could be ectopically expressed in the LE and GE in response to parturition. To address this issue, we used mice in which the *lacZ* gene was introduced into the endogenous *Amhr2* locus (*Amhr2-lacZ*) [18]. In non-pregnant virgins (n = 4) and postpartum *Amhr2-lacZ* females (n = 3 for each experimental group), β-gal expression was only detected in the myometrium; no β-gal expression was detected in the LE or GE (Figure S3). These results suggest that *Amhr2* expression is not altered during homeostasis or by parturition.

### Long-term persistence of stroma-derived endometrial epithelium implicates its functional integration

**Figure 4:**
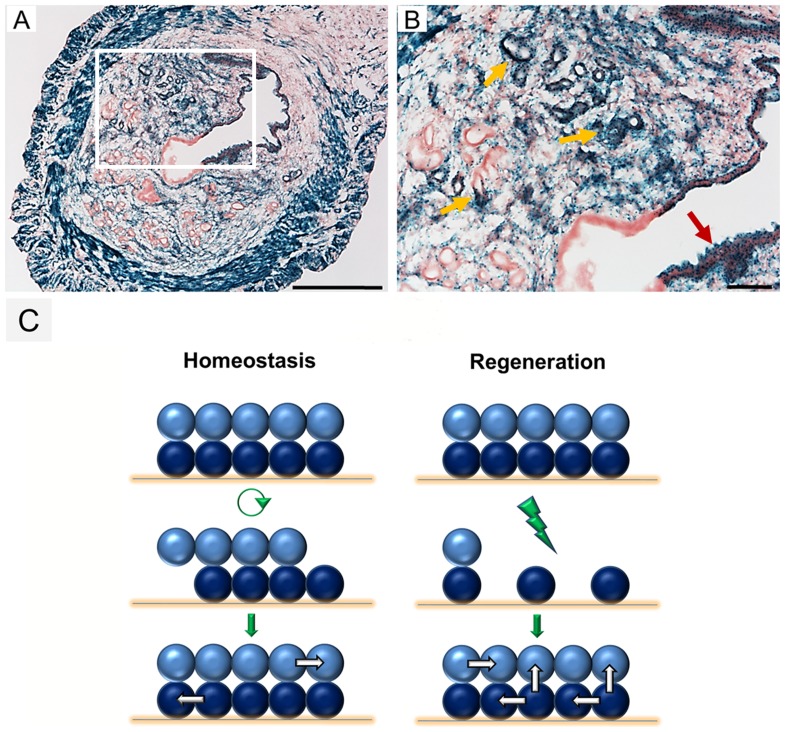
Persistence of stroma-derived epithelial cells and current working models of uterine homeostasis and regeneration. (A) Stroma-derived epithelial cells are detected in the endometrium of *Amhr2-Cre*; *R26R* mice more than two months after parturition. Scale bar  = 100 µm. (B) Higher magnification of Figure 4A, showing stroma-derived luminal (red arrow) and glandular (yellow arrows) epithelium. Scale bar  = 100 µm. (C) Models of uterine homeostasis and postpartum regeneration. A surface epithelial sheet (light blue) and stroma (dark blue) represent the endometrial tissue compartments. Cells lost during estrous cycles are replaced by new cells generated from the same cell compartments. Endometrial damage caused by parturition causes loss of both epithelium and stroma. In addition to stromal cells generating more stroma, some stromal cells sense and respond to the damage and initiate the regeneration process by differentiating into epithelium.

A possible role for stroma-derived epithelial cells would be to transiently assist resident epithelial cells in the re-epithelialization of the uterine lumen during regeneration but turnover perhaps during subsequent estrous cycles. To address this idea, *Amhr2-Cre*; *R26R* females were bred and allowed to give birth. Two months postpartum (i.e. ∼15–20 estrous cycles), the uteri of these mice (n = 3) were examined for β-gal expression. Stroma-derived LE and GE were still clearly present in the endometrium (Figure 4A and 4B). Interestingly, the percentage of β-gal positive LE and GE was relatively higher than the percentage soon after birth. These long-term fate mapping data indicate that once stromal cells differentiate into epithelia, they maintain this epithelial fate and persist during subsequent homeostasis of the uterus.

## Discussion

### Regeneration of the endometrial epithelium and stroma during estrous cycles and postpartum period in the mouse

Although the endometrium undergoes significant cell turnover during the estrous cycle and after parturition, we find that the epithelial cells of the endometrium maintain their fate during these processes. We detected no evidence for an epithelial-to-stromal transition. These results suggest that the epithelial cell compartments of the endometrium are dynamic but are maintained by epithelial lineage-restricted cells. The stromal cell compartment is stable during estrous cycling and apparently the majority of the stroma generates the same tissue after parturition. Surprisingly, we found that a subpopulation of stromal cells derived from *Amhr2*-expressing Müllerian duct mesenchyme can differentiate an epithelial cell fate to form LE and GE upon damage to the endometrium caused by parturition (schematic model in Figure 4C). The epithelial differentiation status of the stroma-derived cells is further supported by the observation that there were initial patches of non-β-gal expressing cells lining the uterine cavity of PPD 1 *Wnt7a-Cre*; *R26R* females, presumably stroma-derived cells undergoing the epithelial transition. By PPD 3, all of the LE and GE were β-gal positive, suggesting induction of epithelial *Wnt7a* expression and therefore *Wnt7a-Cre* activation of Cre reporter expression.

Previous studies have shown that the endometrium contains cells that can give rise to diverse cell types *in vitro* [22], [23]. These studies suggested that the endometrium contains stem cells that are mobilized during regenerative processes. It seems likely that in addition to differentiated stromal cells, *Amhr2-Cre* also marks uterine stem/progenitor cells that reside within the stromal niche. Mesenchymal stem cells have been found in various tissues and can differentiate into diverse cell types using various in vitro induction protocols. Studies using cell-sorting methods have demonstrated that this type of somatic stem cell exists in the stromal compartment of the mammalian endometrium [24], [25]. It is not clear if the stromal stem/progenitor cells that we have identified are mesenchymal stem cells or a distinct cell population. Interestingly, two reports provide evidence that hematopoietic progenitor cells can colonize and differentiate into endometrial epithelium and stroma [26], [27]. Are the uterine stromal/stem progenitor cells that we have identified within the stromal niche the same as the hematopoietic progenitor cells that can differentiate into endometrial epithelium and stroma? This seems less plausible because *Amhr2* has not been shown to be expressed in hematopoietic tissues and its expression ceases in the adult endometrium. Perhaps endometrial homeostasis and regeneration involve lineage-restricted progenitor renewal and differentiation, stromal to epithelial differentiation, and also contributions from hematopoietic progenitor cells.

Mesenchymal-to-epithelial transition (MET) is characterized by the transition of motile spindle or multipolar mesenchymal cells to polarized adhesive epithelial cells. MET and the reverse process epithelial-to-mesenchymal transition (EMT) are two common cellular mechanisms employed during tissue development, regeneration and carcinogenesis [28], [29]. In our system, it is formally possible that the myometrium is the source of the mesenchymal-to-epithelial transition observed because *Amhr2* is active in the myometrium [20]. This could be tested using a Cre mouse line with smooth muscle-restricted expression.

Based on our current results, it appears that only a subset of the *Amhr2-Cre* marked stroma gave rise to epithelial tissue. This may reflect the presence of a subpopulation of stem/progenitor cells or perhaps all stromal cells are capable of becoming epithelial but the inductive signal for regeneration may be limiting. Alternatively, Cre activity may be limiting or mosaic, resulting in regions that do not express the Cre reporter. Regardless, there were large patches of stroma-derived epithelial tissue after parturition. These patches could be derived clonally from single progenitor cells or are the consequence of a coalescence of cells derived from many progenitor cells. Most likely these stroma-derived cells are responding to an extrinsic signal that mobilizes them to participate in the regeneration of the epithelium in concert with resident epithelial cells. These stroma-derived epithelial cells are not just transient assistants for the resident epithelial cells in the regeneration process because they persist at least two months postpartum, experiencing numerous estrous cycles. On the other hand given the observation that the percentage of β-gal positive LE and GE was relatively higher compared to soon after birth, it is possible that the first pregnancy/parturition event created constitutive MET not requiring additional pregnancies.

### Physiological and pathological implications of stroma-derived epithelial cells

Our observations that a subpopulation of uterine stromal cells can respond to the tissue damage of parturition to generate epithelial cell types have implications for human uterine physiology and disease. In women, the *stratum basalis* or deepest layer of the endometrium is preserved after menstruation and contains endometrial gland tissue that is thought to participate in the re-epithelialization of the uterine lumen [30]. There is also evidence that suggests that stromal stem/progenitor cells reside in the *stratum basalis* [31], [32]. We speculate that the dramatic changes in the endometrium that occur during menstruation may also induce a stromal-to-epithelial transition. Based on our results in the mouse model, we hypothesize that there is a constant stream of stroma-derived epithelia incorporated into the LE and GE each month in menstruating women.

The differences of the estrous cycle in mouse (i.e. four to five days) and menstrual cycle in human (i.e. twenty-eight days on average) are significant. In the cycling human endometrium, at least ten days are required for the proliferative phase to regenerate functional layers while there is only minimal morphological distinction among phases in the mouse endometrium. This difference in cycle length along with the extent of cell and tissue damage could be reasons why we did not observe stromal-to-epithelial transition in normal cycling mice. The longer cycling length in menstruating species also implies that their cell-cell adhesions and extracellular matrix in the endometrium would take longer to re-build, leaving a greater chance of the motile stromal cells to invade the epithelial compartment.

Endometriosis is a gynecological condition in which endometrium-like tissues appears ectopically in other organs outside the uterus. Endometriosis causes severe pelvic pain and is one of the common correlates of infertility in women of reproductive age. Retrograde menstruation is one of the ideas to explain the etiology of endometriosis [33]. The subpopulation of uterine stromal cells that can differentiate into epithelial cells could in principle reconstitute functional endometrial tissue if displaced by retrograde menstruation.

The epithelial differentiation ability of endometrial stromal cells also has implications for endometrial cancer. Stromal cells in the *stratum basalis* could accumulate oncogenic genetic lesions that may not be expressed in a stromal differentiation state. However, their transit into an epithelial differentiation state could be permissive for expression and progression of endometrial or other gynecological cancers [34]–[36]. Consistent with this idea, Kim and colleagues [37] recently demonstrated that high-grade serous “ovarian” carcinomas can originate from the stromal compartment of the Fallopian tube by a mesenchymal-to-epithelial transition similar to the regenerative changes that we show in the postpartum uterus.

In summary, we describe a novel stromal-to-epithelial transition during regeneration that is induced by parturition. Our genetic fate-mapping results identify fundamental new insights into the cellular mechanisms of female reproductive tract regeneration. The current study is in a mouse model, a non-menstruating species. Because a large portion of the endometrium is shed and replaced during the menstrual cycle in women, we speculate that this stromal-to-epithelial transition may occur each month in human. This would imply that once a woman begins menstruating the epithelial compartment of her uterus would subsequently be replaced each month by stromal stem/progenitor cells. In the future, it will be interesting to test the idea that endometrial stromal cells carrying *de novo* pathological mutations lead to endometriosis and uterine cancers in human patients [38].

## Materials and Methods

### Mice

*Gt(ROSA)26Sor^tm1Sor^*/J (*R26R*), B6129F1, and B6SJLF1 mice were obtained from the Jackson Laboratory. *Amhr2-Cre* mice were described previously [19]. Mice were analyzed on a C57Bl/6 J (B6), 129/SvEv, SJL/J mixed genetic background. All experimental procedures used for this study were approved by the Institutional Animal Care and Use Committee at the University of Texas M.D. Anderson Cancer Center.

### Generation of *Wnt7a-Cre* bacterial artificial chromosome transgenic mouse

The *Wnt7a-Cre* construct was generated by introducing an IRES-eGFP-Cre FRT neomycin^r^/kanamycin^r^ cassette into the *Wnt7a* locus in a bacterial artificial chromosome RP23-53E13 (The Children's Hospital Oakland – BACPAC Resources) by recombineering (Figure S2). Transgenic mouse were generated by pronuclear microinjection into B6SJLF2 zygotes using standard methods. *Wnt7a-Cre* founders were screened by allele-specific PCR using primers: *Wnt7a-Cre* P1 (5′ – ctgttcagatgactgcagtactagc – 3′), P2 (5′ – gccttattccaagcggcttcg – 3′), P3 (5′ – ccttcttgacgagttcttctgagg – 3′) and P4 (5′ – gccttacaagttgaatgcacgtgtg – 3′). PCR reactions were performed with a melting temperature of 95°C for 45 seconds, an annealing temperature of 55°C for 45 seconds and an extension temperature of 72°C for 45 seconds for 35 cycles. P1+P2 produce a product of 735 bp and P3+P4 a product of 543 bp. The *Wnt7a-Cre* transgenic mouse line was maintained by backcrossing to B6 mice.

### Tissue processing

Non-pregnant and postpartum females were sacrificed and their uteri were fixed in 4% paraformaldehyde (PFA) in phosphate buffer saline (PBS) at 4°C overnight. For frozen sections, uterine tissue was cryoprotected with 30% sucrose for at least 24 h and embedded in 30% sucrose/OCT (Tissue-Tek). For paraffin sections, fixed uterine tissue was washed in PBS 3 times and stored in 70% ethanol at 4°C. Tissues were embedded in paraffin wax. 10 µm frozen sections and 4 µm paraffin sections were cut. Sections were collected across the rostral–caudal axis of the uterus on Superfrost Plus slides (Fisher).

### X-gal staining

Freshly cut frozen sections were washed three times in PBS containing 0.02% of NP-40 and 0.01% of sodium deoxycholate. Sections were then incubated at 37°C in X-gal staining solution, containing 5 mM K_3_Fe(CN)_6_, 5 mM K4Fe(CN)6, 0.02% NP-40, 0.01% sodium deoxycholate, 2 mM MgCl2, 5 mM EGTA and X-gal (1 mg/ml) in PBS. After X-gal staining, sections were washed in PBS three times, counterstained with Nuclear Fast Red (Vector Laboratories Inc.), dehydrated, and immersed in HistoClear (National Diagnostics). Cover slips were mounted using a resin-based mounting medium.

### Cell proliferation and death assays

BrdU was administrated by intraperitoneal injection to postpartum mice at a concentration of 50 mg/kg, 2 hours before sacrificing the animals. BrdU immunostaining was adapted from the protocol provided by Calbiochem. Briefly, paraffin sections were first de-waxed and heated in 0.01% sodium citrate buffer (pH 6.0) for 10 min for epitope exposure. Samples were incubated in denaturing solution for 30 min and after a rinse in PBS incubated in blocking solution for 10 min. Sections were then incubated with biotinylated mouse anti-BrdU primary antibody (1∶200 dilution) at room temperature for 1 hr and secondary antibody conjugated with streptavidin-peroxidase for 10 min. After washing in PBS 3 times, AEC solution was applied to sections, and color reactions were observed under the microscope. Samples were then sealed in glycerol-based mounting medium.

For TUNEL, paraffin sections were deparaffinized and incubated in proteinase K solution (20 mg/ml in 10 mM Tris-HCl (pH 7.4–8) for 20 min at 37°C. After 3 washes in PBS, sections were permeabilized with 0.1% Triton X-100 in 0.1% sodium citrate for 2 min on ice, and then in a 50 ml TUNEL reaction mixture (Roche Applied Science) for 1 hr at 37°C in the dark. Samples were then washed in PBS 3 times, stained with To-Pro-3 (Invitrogen) and covered with Vectashield mounting medium (Vector Laboratories Inc.).

For BrdU staining and TUNEL assay, negative controls are shown in Figure S4.

### Immunofluorescence

Indirect immunofluorescence was performed on frozen sections. In brief, sections were dried at room temperature for 20 min and washed 3 times in PBS. Sections were incubated in blocking solution containing 5% normal goat serum and 3% bovine serum albumin in PBS for 30 min at room temperature. Goat anti-β-gal antibody (1∶300, MP Biomedical) and rat anti-cytokeratin antibody (1∶300, TROMA I, Developmental Studies Hybridoma Bank) were applied to samples and incubated overnight at 4°C. Sections were washed in PBS 3 times and incubated with fluorophore-conjugated goat anti-rabbit (for β-gal) and donkey anti-rat (for cytokeratin) IgG solution for 30 min at room temperature. Samples were then washed in PBS 5 times and covered with DAPI-containing Vectashield mounting medium. No immunoreactivity against β-gal or cytokeratin was detected in negative control samples lacking incubation with primary antibody (Figure S4).

## Supporting Information

Figure S1 **Endometrial cell proliferation and cell death during estrous cycles in the mouse.** Cell proliferation marked by BrdU labeling at estrus (A) and metestrus (B). At estrus, BrdU-positive signals are prominent in the luminal epithelium (arrow); some proliferating cells are also found in the stroma and fewer cells were labeled in the glandular epithelium. At metestrus, the number of BrdU-positive cells was generally low, but a few cells were detectable in the luminal epithelium, glandular epithelium and stroma. Cell death marked by the TUNEL assay at estrus (C) and metestrus (D). TUNEL-positive signals are very low at estrus but prominent in the luminal epithelium (arrow) and some cells in the stroma at metestrus. Scale bars in A–D = 100 µm. Scale bars in boxed areas of A and B = 10 µm; of C and D = 12.5 µm.
(TIF)

Figure S2 **Generation and characterization of** ***Wnt7a-Cre*** **BAC transgenic mice.** (A) Generation *Wnt7a-Cre* BAC transgenic mice. *Wnt7a* consists of 4 exons (boxes) with both translated (blue) and untranslated (unfilled) regions. An IRES-eGFP-Cre FRT flanked neo^r^/kan^r^ (neo/kan) expression cassette was introduced into exon 2 of a BAC clone by recombineering in bacteria. Correct targeting of the BAC and screening of founders were analyzed by PCR. P1, primer 1; P2, primer 2; P3, primer 3; P4, primer 4. (B) Whole mount β-gal staining of an E13.5 *Wnt7a-Cre*; *R26R-lacZ* urogenital ridge. Cre reporter expression is restricted to the Müllerian duct epithelium (MDE, arrow). (C) Transverse section showing Cre reporter expression limited to the Müllerian duct epithelium. G: gonad; WD: Wolffian duct. Scale bars  = 500 µm in B; 50 µm in C.
(TIF)

Figure S3 **β-gal expression in the uterus of non-pregnant and postpartum** ***Amhr2 ^lacZ/+^*** **females.** (A–H), non-pregnant, PPD 0, 1, and 3 uteri, showing β-gal expression limited to the myometrium but not in the LE or GE. (B, D, F, and H) are higher magnification images of A, C, E, and G, respectively. Black arrows point to sparse β-gal positive cells in the myometrium. Scale bars in A, C, E and G = 50 µm; in B, D, F and H = 100 µm.
(TIF)

Figure S4 **Negative controls for BrdU staining, immunefluorescence and TUNEL assay.** (A–B) Diaminobenzidine (DAB)-assisted immunohistochemistry against BrdU on the uterine section from a BrdU-injected mouse. The section is not incubated with primary antibody against BrdU. (C–F) Uterine section from a wildtype mouse stained with (C) DAPI, (D) Alexa 488-conjugated goat anti-rabbit and (E) Alexa 594-conjugated donkey anti-rat IgG solution. Fluorescent signal of RGB channel-merge is shown in (F). (G–I) Uterine sample stained with Topro3 (G) and incubated with TUNEL-label solution (H, without terminal transferase). Merged image of red and green channels is shown in (I). Scale bars  = 100 µm.
(TIF)
