# Supplementary figures and images for: Stromal-to-Epithelial Transition during Postpartum Endometrial Regeneration
Source: PLoS One. 2012 Aug 27;7(8):e44285. doi: 10.1371/journal.pone.0044285 (PMC3433810; doi:10.1371/journal.pone.0044285)

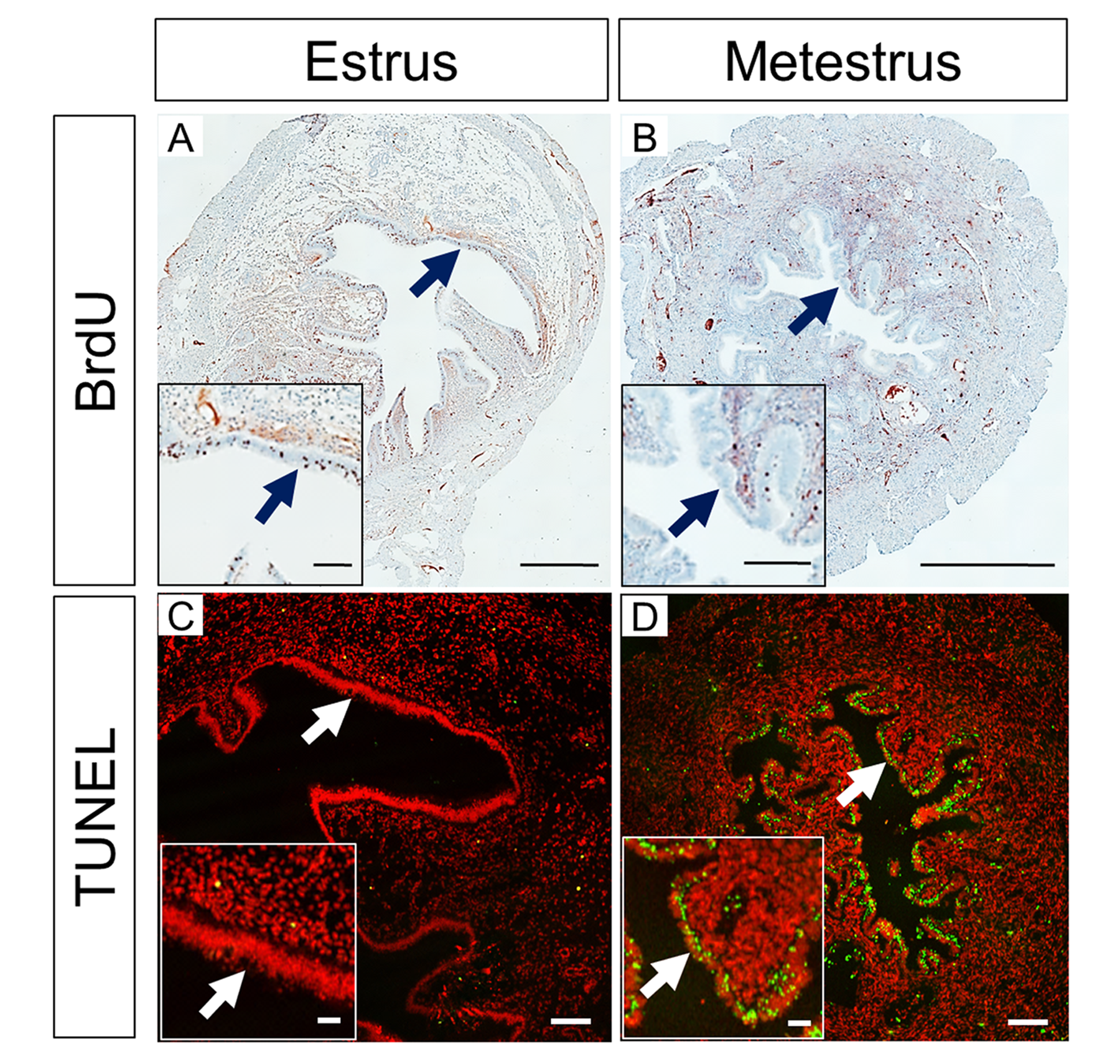

Supplement: Figure S1 — Endometrial cell proliferation and cell death during estrous cycles in the mouse. Cell proliferation marked by BrdU labeling at estrus (A) and metestrus (B). At estrus, BrdU-positive signals are prominent in the luminal epithelium (arrow); some proliferating cells are also found in the stroma and fewer cells were labeled in the glandular epithelium. At metestrus, the number of BrdU-positive cells was generally low, but a few cells were detectable in the luminal epithelium, glandular epithelium and stroma. Cell death marked by the TUNEL assay at estrus (C) and metestrus (D). TUNEL-positive signals are very low at estrus but prominent in the luminal epithelium (arrow) and some cells in the stroma at metestrus. Scale bars in A–D = 100 µm. Scale bars in boxed areas of A and B = 10 µm; of C and D = 12.5 µm. (TIF) [file pone.0044285.s001.tif]

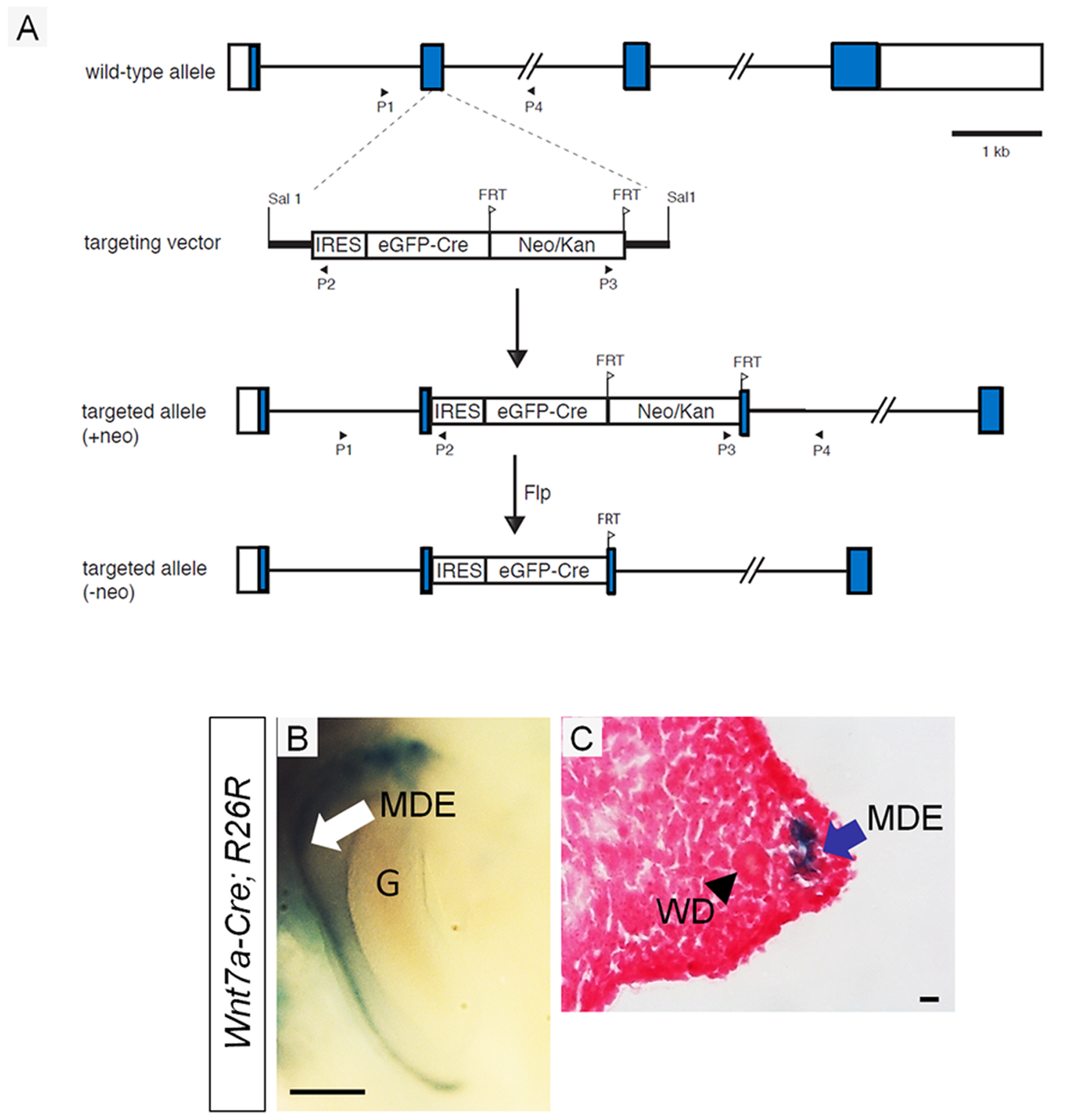

Supplement: Figure S2 — Generation and characterization of Wnt7a-Cre BAC transgenic mice. (A) Generation Wnt7a-Cre BAC transgenic mice. Wnt7a consists of 4 exons (boxes) with both translated (blue) and untranslated (unfilled) regions. An IRES-eGFP-Cre FRT flanked neor/kanr (neo/kan) expression cassette was introduced into exon 2 of a BAC clone by recombineering in bacteria. Correct targeting of the BAC and screening of founders were analyzed by PCR. P1, primer 1; P2, primer 2; P3, primer 3; P4, primer 4. (B) Whole mount β-gal staining of an E13.5 Wnt7a-Cre; R26R-lacZ urogenital ridge. Cre reporter expression is restricted to the Müllerian duct epithelium (MDE, arrow). (C) Transverse section showing Cre reporter expression limited to the Müllerian duct epithelium. G: gonad; WD: Wolffian duct. Scale bars = 500 µm in B; 50 µm in C. (TIF) [file pone.0044285.s002.tif]

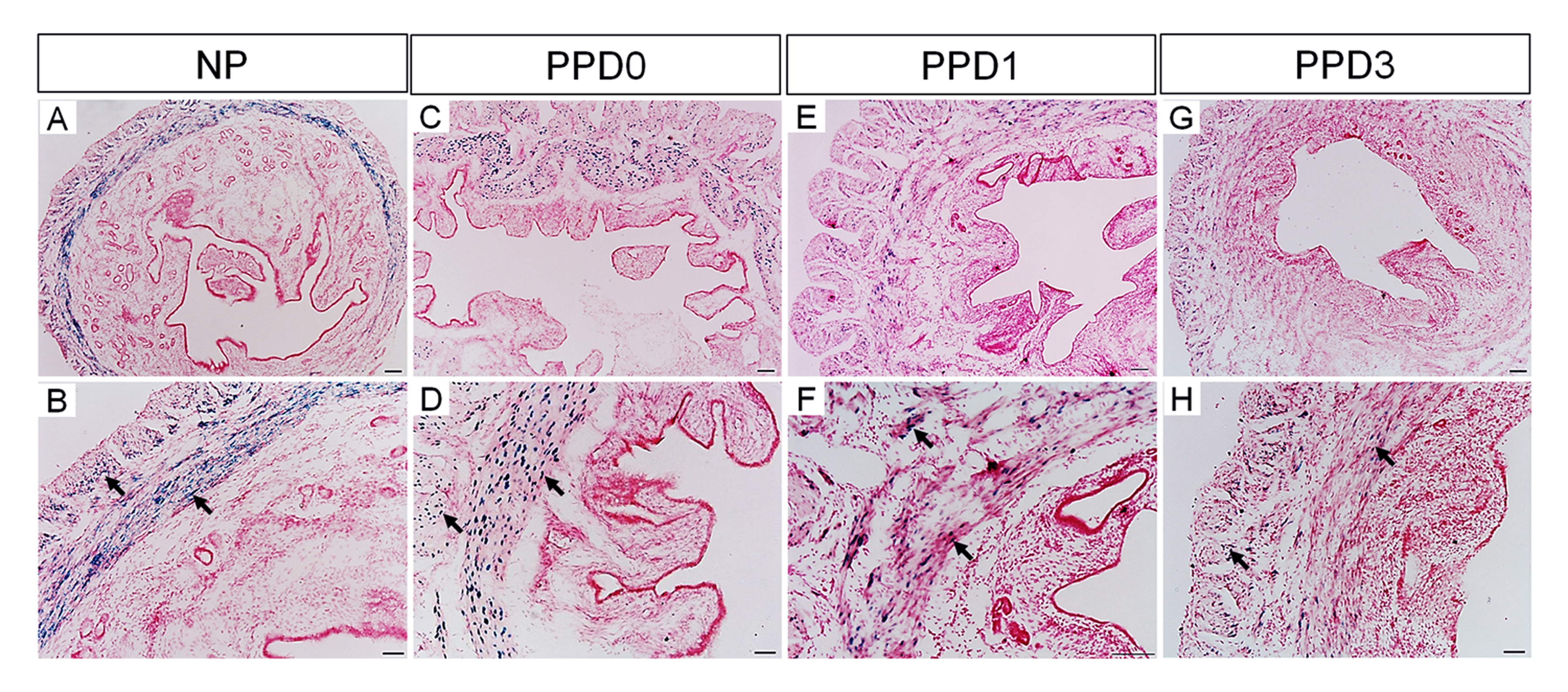

Supplement: Figure S3 — β-gal expression in the uterus of non-pregnant and postpartum Amhr2 lacZ/+ females. (A–H), non-pregnant, PPD 0, 1, and 3 uteri, showing β-gal expression limited to the myometrium but not in the LE or GE. (B, D, F, and H) are higher magnification images of A, C, E, and G, respectively. Black arrows point to sparse β-gal positive cells in the myometrium. Scale bars in A, C, E and G = 50 µm; in B, D, F and H = 100 µm. (TIF) [file pone.0044285.s003.tif]

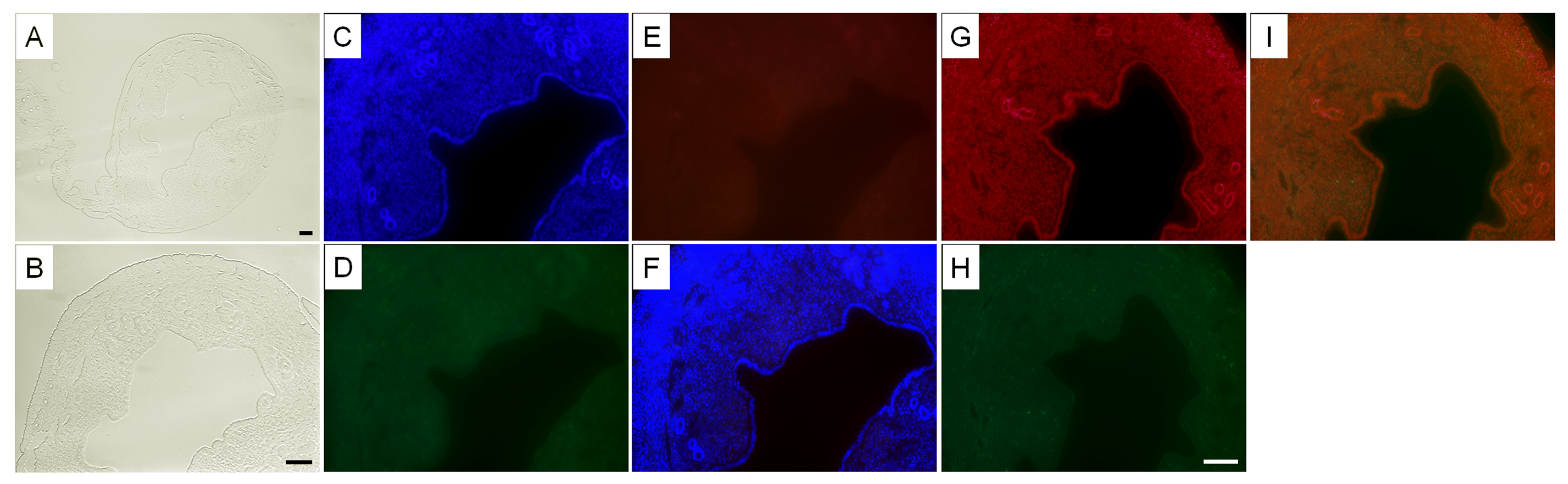

Supplement: Figure S4 — Negative controls for BrdU staining, immunefluorescence and TUNEL assay. (A–B) Diaminobenzidine (DAB)-assisted immunohistochemistry against BrdU on the uterine section from a BrdU-injected mouse. The section is not incubated with primary antibody against BrdU. (C–F) Uterine section from a wildtype mouse stained with (C) DAPI, (D) Alexa 488-conjugated goat anti-rabbit and (E) Alexa 594-conjugated donkey anti-rat IgG solution. Fluorescent signal of RGB channel-merge is shown in (F). (G–I) Uterine sample stained with Topro3 (G) and incubated with TUNEL-label solution (H, without terminal transferase). Merged image of red and green channels is shown in (I). Scale bars = 100 µm. (TIF) [file pone.0044285.s004.tif]
